# Transcriptomic analysis of the hepatic response to stress in the red cusk-eel (*Genypterus chilensis*): Insights into lipid metabolism, oxidative stress and liver steatosis

**DOI:** 10.1371/journal.pone.0176447

**Published:** 2017-04-27

**Authors:** Sebastian Naour, Brisa M. Espinoza, Jorge E. Aedo, Rodrigo Zuloaga, Jonathan Maldonado, Macarena Bastias-Molina, Herman Silva, Claudio Meneses, Cristian Gallardo-Escarate, Alfredo Molina, Juan Antonio Valdés

**Affiliations:** 1 Universidad Andres Bello, Laboratorio de Biotecnología Molecular, Facultad Ciencias Biológicas, Santiago, Chile; 2 Interdisciplinary Center for Aquaculture Research (INCAR), Concepción, Chile; 3 Universidad de Chile, Facultad de Ciencias Agronómicas, Departamento de Producción Agrícola, Laboratorio de Genómica Funcional & Bioinformática, Av. Santa Rosa, La Pintana, Santiago, Chile; 4 Universidad Andres Bello, Centro de Biotecnología Vegetal, FONDAP Center for Genome Regulation, Facultad de Ciencias Biológicas, Santiago, Chile; 5 Universidad de Concepción, Laboratory of Biotechnology and Aquatic Genomics, Concepción, Chile; 6 Universidad Andres Bello, Centro de Investigación Marina Quintay (CIMARQ), Facultad de Ecología y Recursos Naturales, Valparaíso, Chile; National Cheng Kung University, TAIWAN

## Abstract

Teleosts exhibit a broad divergence in their adaptive response to stress, depending on the magnitude, duration, and frequency of stressors and the species receiving the stimulus. We have previously reported that the red cusk-eel (*Genypterus chilensis*), an important marine farmed fish, shows a physiological response to stress that results in increased skeletal muscle atrophy mediated by over-expression of components of the ubiquitin proteasome and autophagy-lysosomal systems. To better understand the systemic effects of stress on the red cusk-eel metabolism, the present study assessed the transcriptomic hepatic response to repetitive handling-stress. Using high-throughput RNA-seq, 259 up-regulated transcripts were found, mostly associated with angiogenesis, gluconeogenesis, and triacylglyceride catabolism. Conversely, 293 transcripts were down-regulated, associated to cholesterol biosynthesis, PPARα signaling, fatty acid biosynthesis, and glycolysis. This gene signature was concordant with hepatic metabolite levels and hepatic oxidative damage. Moreover, the increased plasmatic levels of AST (aspartate aminotransferase), ALT (alanine aminotransferase) and AP (alkaline phosphatase), as well as liver histology suggest stress-induced liver steatosis. This study offers an integrative molecular and biochemical analysis of the hepatic response to handling-stress, and reveals unknown aspects of lipid metabolism in a non-model teleost.

## Introduction

Stress generated during intensive fish farming is a major factor negatively affecting animal growth and health, and results in a decline in finfish aquaculture production [1,2]. Among the main types of stressors are those induced by physical stimuli, such as transport, capture, or handling [3,4], which are unavoidable in intensive aquaculture [5]. Fish possess a set of physiological strategies, allowing them to respond to stressors through adaptive neuroendocrine adjustments, collectively termed the stress response [6]. In fish, this stress response is mediated by the hypothalamic-pituitary-interrenal (HPI) axis, which is responsible for promoting the cortisol synthesis and secretion through interrenal cells into the bloodstream [7].

The liver is one of the most important target organs in the adaptive response to stress, and is a fundamental metabolic tissue for energy substrate administration [8], because hepatic tissue can synthesize *de novo* glucose for non-hepatic tissues during periods of stress [9]. In addition, the liver is a key tissue involved in somatic growth regulation, the immune response, and detoxification [10–13]. Recent advances in transcriptomic technologies now allow a comprehensive understanding of how physical disturbances modulate metabolic adaptations to stress [14,15]. While early studies were conducted on a limited number of species using microarray technology [16–18], it is now possible, thanks to progress in high-throughput sequencing technologies, to study the adaptive function of liver tissue in a range of commercially important species, including channel catfish (*Ictalurus punctatus*) [19], rainbow trout (*Oncorhynchus mykiss*) [20], and large yellow croaker (*Larimichthys crocea*) [21], among others. Such studies demonstrate that each species has different adaptive mechanisms and molecular responses to stress, revealing particular details in their species-specific stress tolerances, meaning that culturing a new species requires an integrative and detailed knowledge of tissue-specific stress responses.

The red cusk-eel (*Genypterus chilensis*) is a new economically important marine species for the Chilean aquaculture industry [22], but low handling-stress tolerance results in high mortality rates during juvenile stages [23]. Handling-stress facilitates red cusk-eel skeletal muscle atrophy through the coordinated expression of components of the ubiquitin-proteasome and autophagy-lysosome systems, revealing particularities in the compensatory response to stress [24]. Therefore, in the present study, we evaluate the hepatic response to handling-stress in the red cusk-eel using RNA-seq analysis, using a recently reported hybrid reference transcriptome [25]. This transcriptomic analysis was complemented by a quantification of hepatic metabolites, plasmatic enzyme activities, liver histology and oxidative stress markers. Our results strongly suggest that handling-stress has detrimental effects in this species, modulating the expression of genes associated with hepatic lipid metabolism and inducing liver steatosis.

## Material and methods

### Ethics statement

The study adhered to animal welfare procedures, and was approved by the bioethical committees of the Universidad Andres Bello and the National Commission for Scientific and Technological Research (CONICYT) of the Chilean government. The field studies did not involve endangered or protected species. The activities were performed at the Centro de Investigación Marina de Quintay and authorized by the Andres Bello University.

### Kit manufacturer recommendations

All kits were applied following manufacturer recommendations, unless noted.

### Animals and experimental design

Juvenile red cusk-eels (*Genypterus chilensis*) with an average weight of 900 ± 50 g and length of 55 ± 5 cm were collected from the Centro de Investigación Marina de Quintay (CIMARQ) (33°13′S 71°38′W, Valparaíso Region, Chile). Eight fish were kept under natural temperature and light:dark photoperiod conditions (13°C ± 1°C and L:D 12:12) for the spring season. The animals were then split into control and stressed groups, placed in separate 90 L tanks, and acclimated for two weeks before the handling-stress protocol. Fish were fed once daily with 6-mm commercial pellets, containing 60% protein, 6% lipids, 11% carbohydrates, 15% ashes, and 8% humidity (Skretting, Puerto Montt, Chile). The stressed group was then subjected to a standardized handling-stress protocol consisting of netting and chasing the fish for five minutes daily for five days. The management bias from adjacent tanks was prevented through the completely blocked by black covers on all tanks. Six hours after the final handling stimulation, the four stressed and four control fish were captured and anaesthetized (3-aminobenzoic acid ethyl ester, 100 mg/L^-1^). Blood was collected from the caudal vein with 1 ml heparinized (10 mg/mL) syringes and centrifuged at 5000 xg for 10 min at 4°C to obtain plasma, frozen in liquid nitrogen, and stored at -80°C. The fish were then euthanized through an overdose of anesthetic (3-aminobenzoic acid ethyl ester, 300 mg/L). Livers were collected and immediately frozen in liquid nitrogen and stored at -80°C.

### AST, ALT and AP measurements

The plasmatic activity of AST (aspartate aminotransferase), ALT (alanine aminotransferase) and AP (alkaline phosphatase) were determined using commercially available kits from Valtek (Santiago, Chile). The enzymatic activity of AST, ALT, and AP are determined, respectively, by the production of colorimetric products from the generation of glutamate (colorimetric product: 450 nm), pyruvate (535 nm), and p-nitrophenol (405 nm).

### Hepatic metabolites

The hepatic metabolite levels for glycogen, triglyceride, non-esterified free fatty acid (NEFA) and total cholesterol were determined using commercially available kits from Cell Biolabs (CA, USA). Ultrasonic disruption in PBS containing 0.5% Triton X-100 was used to homogenize 200 mg of liver tissue, which was centrifuged at 5000 xg for 10 minutes at 4°C. The supernatant was collected, stored on ice, and used for glycogen and triglyceride quantification using the Glycogen Assay Kit (Cell Biolabs), and Serum Triglyceride Quantification Kit (Cell Biolabs), respectively. For cholesterol and free fatty acid quantification [26], 10 mg of liver tissue was extracted with 200 μL of a chloroform:isopropanol:NP-40 (7:11:0.1) mixture in a micro-homogenizer. The extract was centrifuged at 15,000 xg for 10 minutes, and the organic phase was transferred to a new tube and air dried at 50ºC to remove the chloroform. The dried lipids were dissolved and homogenized in 200 μL of 1X Assay Diluent via vortexing. Cholesterol and NEFA were quantified using Total Cholesterol Assay Kit (Cell Biolabs) and Free Fatty Acid Assay Kit (Cell Biolabs), respectively.

### Hepatic oxidative stress assays

DNA oxidative damage, protein carbonylation, and lipid peroxidation were determined using commercially available kits from Cell Biolabs (CA, USA). For oxidative DNA damage assessment, genomic DNA (gDNA) was extracted from 25 mg of liver using the Isolate II Genomic DNA Kit (Bioline, MA, USA), and dissolved in TE buffer. DNA was quantified by spectrophotometry using NanoDrop with the Epoch Multi-Volume Spectrophotometer System (BioTek, VT, USA). Assessment of gDNA quality and visualization were performed by electrophoresis on a 1% agarose gel in TAE 1X containing ethidium bromide. In order to determine apurinic/apyrimidinic (AP) sites, gDNA was mixed with the aldehyde reactive prove (ARP) reaction using the OxiSelect Oxidative DNA Damage Quantification Kit (Cell Biolabs, CA, USA), reading absorbance at 450 nm. For assessment of protein carbonylation, total proteins were extracted from 100 mg of liver in 1 ml of lysis buffer containing 50 mM Tris-HCl pH 7.4, 150 mM NaCl, 1 mM EDTA, and 1% NP-40, and solubilized at 4°C after 12,000 xg centrifugation. Protein concentration was determined using a Pierce® BCA Protein Assay Kit (Thermo Scientific, IL, USA). Then, protein carbonyl content was quantified by the OxiSelect Protein Carbonyl Spectrophotometric Assay (Cell Biolabs, CA, USA). Lipid peroxidation assessment was quantified by the formation of thydroxynonenal (HNE) protein adducts using the OxiSelect HNE Adduct Competitive ELISA Kit (Cell Biolabs, CA, USA).

### Liver histology

The livers of the red cusk-eel were dissected immediately after euthanization according to the protocol described previously. The livers were fixed in 4% paraformaldehyde, embedded in paraffin, and sectioned at a 5 μM thickness of three slices per liver. The samples were stained with hematoxylin/eosin, observed in a Olympus BX-61 microscope and photographed with a Leica DF300 camera.

### Liver RNA sequencing

Total RNA was extracted using the RNeasy Mini Kit (Qiagen, TX, USA). RNA was quantified spectrophotometrically using NanoDrop with the Epoch Multi-Volume Spectrophotometer System (BioTek, VT, USA). Total RNA isolated from the liver was treated with DNase I to remove genomic DNA. RNA concentration was measured using a Qubit® 2.0 Fluorometer (Life Technology, Carlsbad, CA, USA), and RNA integrity was determined using the Fragment Analyzer™ Automated CE System (Analytical Advanced Technologies, Ames, IA, USA). Equal quantities of total RNA from four fish were pooled by condition, and the total RNA was used to prepare mRNA libraries. Complementary DNA (cDNA) libraries were constructed using TruSeq RNA Sample Preparation kit v2 (Illumina®, USA). Libraries were sequenced (2 x 250bp) with the MiSeq (Illumina®) platform at the Centro de Biotecnología Vegetal (UNAB, Chile).

### Analysis and representation of differentially expressed transcripts

Raw sequencing reads were trimmed by removing adaptor sequences, low quality sequences (quality scores less than 10), and sequences with lengths less than 30 bp. To identify differentially expressed transcripts, the reads from control and stressed conditions were mapped to the *G*. *chilensis* reference transcriptome [25], using CLC Genomics Workbench, v.7.0.3 (http://www.clcbio.com), with the following parameters: mismatches = 2, minimum fraction length = 0.9, minimum fraction similarity = 0.8, and maximum hits per read = 5. Gene expressions were based on reads per kilobase of exon model per million mapped read (RPKM) values. Transcripts with absolute fold-change values > 2.0 and FDR corrected P value < 0.05 were included in the GO and KEGG enrichment analyses.

The DAVID database [27] was used for the analysis of up- and down-regulated transcripts and functionally related gene cluster identification [24]. Cut-off enrichment scores of 4.0 or greater were considered for analysis. The construction of map pathways for Angiogenesis (WP1539_79949), Cholesterol Biosynthesis (WP197_78758), PPAR Pathway (WP2878_7968), Fatty Acid Biosynthesis (WP357_70641), Glycolysis and Gluconeogenesis (WP534_78585), and Triacylglyceride Synthesis (WP325_71223) were performed using PathVisio, v.3.0.

### RNA-seq validation by real-time qPCR

All quantitative real-time polymerase chain reaction (qPCR) assays were carried out complying with the MIQE guidelines [28]. Total RNA was extracted from liver tissue using the RNeasy Mini Kit (Qiagen, TX, USA). RNA was quantified by spectrophotometry using NanoDrop with the Epoch Multi-Volume Spectrophotometer System (BioTek, VT, USA). Only RNA with an A260/280 ratio between 1.9 and 2.1 were used for cDNA synthesis. Residual genomic DNA was removed using the genomic DNA wipeout buffer included in the Quantitect Reverse Transcription Kit (Qiagen, TX, USA). Subsequently, 1 μg of RNA was reverse transcribed into cDNA for 30 min at 42°C.

The qPCR assessments were performed using a Stratagene MX3000P qPCR system (Stratagene, La Jolla, CA, USA). Each reaction mixture contained 7.5 μl of 2× Brilliant® II SYBR® master mix (Stratagene, La Jolla, CA, USA), 6 μl of cDNA (40-fold diluted) and 250 nM of each primer in 20 μl final volume. The list of primers and amplification efficiencies are described in S1 Table. Amplifications were performed in triplicate following thermal cycling conditions of: initial activation of 10 minutes at 95°C, followed by 40 cycles of 30s of denaturation at 95°C, 30s of annealing at 54–60°C, and 30s of elongation at 72°C. In order to confirm the presence of a single PCR product, dissociation curve analysis of the PCR products was performed and evaluated by electrophoresis on a 2% agarose gel. With the purpose of calculating the assay’s efficiency, 2-fold dilution series were created from a cDNA pool. Efficiency values were estimated from the slope of the efficiency curve: E = 10(-1/slope)^-1^. The reference gene, 40S ribosomal protein S30 (*fau*), was used for gene expression normalization, and control reactions included a no-template control and a no-reverse-transcriptase control. QGene was used for analysis of gene expression [29].

### Statistical analysis

Data are expressed as the mean ± SEM. Differences in means between groups were determined using one-way ANOVAs, followed by a Bonferroni post hoc test. Data were accepted as significant at a value of P < 0.05. Correlations between RNA-seq and qPCR data were assessed through multiple linear regression, using coefficients of determination (R^2^) and p-values. All statistical analyses were performed using GraphPad Prism, v.5.00 (GraphPad Software, CA, USA).

## Results

### Assessment of hepatic metabolites

**Fig 1:**
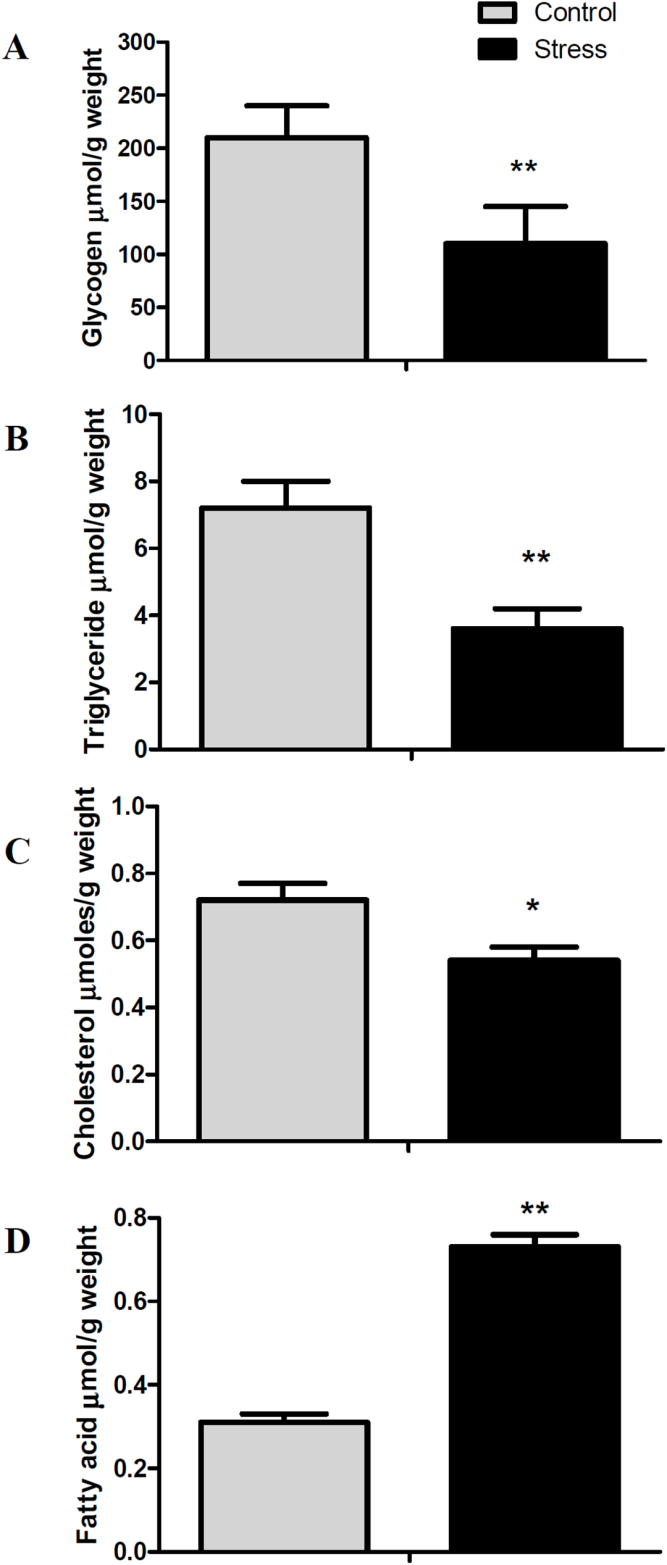
Hepatic metabolites levels of glycogen, triglyceride, cholesterol and non-esterified fatty acids (NEFA). All data are represented as means ± SEM (n = 4). Significant differences between control and stressed groups are shown as * (P < 0.05) and ** (P < 0.01).

We previously reported that plasmatic cortisol and glucose levels significantly increased after 5 days of daily handling-stress [24]. Considering how the liver plays a central role in the metabolic homeostasis of carbohydrates and lipids, we measured the hepatic metabolite levels of glycogen, triglyceride, cholesterol and non-esterified fatty acids (NEFA). We found decreased glycogen (Fig 1A), triglyceride (Fig 1B) and cholesterol levels (Fig 1C) in comparison to control conditions. Conversely, the levels of NEFA were higher than in the control condition (Fig 1D). These results indicate a dynamic response to stress-mediated carbohydrate and lipid metabolism.

### Hepatic transcriptomic responses of red cusk-eel to handling-stress

To understand how stress modulates the hepatic stress response in a comprehensive manner, RNA-seq analyses were performed on liver samples from control and stressed groups including cDNA library replicates of each condition. We obtained a total of 12,950,452 paired-end reads for the control and a total of 13,669,452 paired-end reads for the stressed group. Raw reads have been deposited in the NCBI Sequence Read Archive under the study accession number SRX1056881. After sequence trimming for adapter subtraction and filtering of low quality base pairs, the data sets were reduced to 11,912,668 and 12,270,950 high-quality reads, respectively. Differentially expressed transcripts (DETs) were estimated by FPKM, mapping obtained reads to the reported reference transcriptome for *G*. *chilensis* [25], resulting in a mapping of roughly 98.5% of the reads. A total of 552 DETs showed significant differential expression between both conditions. Of these, there were 259 up-regulated transcripts and 293 down-regulated transcripts in stressed fish. A complete list of the differentially expressed transcripts is included in S2 Table.

### GO cluster enrichment and pathway analysis

The up- and down-regulated genes were analyzed through the DAVID database and clustered based on GO terms. Table 1 summarizes the top four clusters of functional categories with Enrichment Score > 4 up-regulated under stress. The cluster with the highest enrichment scores (ES 6.69) includes biological processes like vasculature development, blood vessel development, blood vessel morphogenesis, and angiogenesis. Other enriched clusters up-regulated by stress included biological processes like cell migration, molecular function like heparin binding and biological process like response to hormone stimulus. Table 2 summarizes the top four clusters of functional categories with Enrichment Score > 4 down-regulated under stress. The cluster with the highest enrichment scores (ES 17.02) includes biological processes like steroid metabolic process, sterol metabolic process, sterol biosynthetic process, cholesterol metabolic process, cholesterol biosynthetic process, steroid biosynthetic process, lipid biosynthetic process. Other enriched clusters down-regulated by stress included cellular component like endoplasmic reticulum, molecular function like monocarboxylic acid binding and cellular component like microsome.

**Table 1:** Over-represented functional categories in clusters of up-regulated transcripts in response to handling stress.

GO category	GO Term	GO ID	Genes Count	P_Value
**Annotation Cluster 1**	**Enrichment Score: 6.69**			
Biological Process	vasculature development	GO:0001944	21	2.1E-9
Biological Process	blood vessel development	GO:0001568	20	8.6E-9
Biological Process	blood vessel morphogenesis	GO:0048514	17	1.8E-7
Biological Process	angiogenesis	GO:0001525	10	5.0E-4
**Annotation Cluster 2**	**Enrichment Score: 4.24**			
Biological Process	cell migration	GO:0016477	16	2.7E-5
Biological Process	cell motion	GO:0006928	21	5.1E-5
Biological Process	cell motility	GO:0048870	16	9.0E-5
Biological Process	localization of cell	GO:0051674	16	9.0E-5
**Annotation Cluster 3**	**Enrichment Score: 4.16**			
Molecular Function	heparin binding	GO:0008201	11	2.0E-6
Molecular Function	glycosaminoglycan binding	GO:0005539	11	3.1E-5
Molecular Function	polysaccharide binding	GO:0030247	11	7.1E-5
Molecular Function	pattern binding	GO:0001871	11	7.1E-5
Molecular Function	carbohydrate binding	GO:0030246	13	4.8E-3
**Annotation Cluster 4**	**Enrichment Score: 3.74**			
Biological Process	response to hormone stimulus	GO:0009725	19	1.7E-5
Biological Process	response to endogenous stimulus	GO:0009719	20	1.8E-5
Biological Process	response to organic substance	GO:0010033	27	5.3E-5
Biological Process	response to steroid hormone stimulus	GO:0048545	12	2.0E-4
Biological Process	cellular response to hormone stimulus	GO:0032870	9	1.1E-3
Biological Process	response to peptide hormone stimulus	GO:0043434	8	1.0E-2

**Table 2:** Over-represented functional categories in clusters of down-regulated transcripts in response to handling stress.

GO category	GO Term	GO ID	Genes Count	P_Value
**Annotation Cluster 1**	**Enrichment Score: 17.02**			
Biological process	steroid metabolic process	GO:0008202	31	1.2E-19
Biological process	sterol metabolic process	GO:0016125	24	1.2E-19
Biological process	sterol biosynthetic process	GO:0016126	17	1.4E-19
Biological process	cholesterol metabolic process	GO:0008203	21	8.6E-17
Biological process	cholesterol biosynthetic process	GO:0006695	14	8.8E-17
Biological process	steroid biosynthetic process	GO:0006694	20	3.0E-16
Biological process	lipid biosynthetic process	GO:0008610	33	1.4E-15
**Annotation Cluster 2**	**Enrichment Score: 7.38**			
Cellular Component	endoplasmic reticulum	GO:0005783	53	4.0E-13
Cellular Component	endoplasmic reticulum part	GO:0044432	28	1.5E-10
Cellular Component	nuclear envelope-endoplasmic reticulum network	GO:0042175	21	2.2E-7
Cellular Component	endoplasmic reticulum membrane	GO:0005789	20	4.2E-7
Cellular Component	organelle membrane	GO:0031090	43	2.3E-6
Cellular Component	endomembrane system	GO:0012505	29	4.1E-4
**Annotation Cluster 3**	**Enrichment Score: 6.45**			
Molecular Function	monocarboxylic acid binding	GO:0033293	11	2.3E-8
Molecular Function	carboxylic acid binding	GO:0031406	16	2.5E-8
Molecular Function	fatty acid binding	GO:0005504	9	2.1E-7
Molecular Function	acyl-CoA binding	GO:0000062	5	1.3E-4
**Annotation Cluster 4**	**Enrichment Score: 4.45**			
Cellular Component	microsome	GO:0005792	20	5.8E-8
Cellular Component	vesicular fraction	GO:0042598	20	9.2E-8
Cellular Component	cell fraction	GO:0000267	39	5.3E-5
Cellular Component	membrane fraction	GO:0005624	25	1.1E-2
Cellular Component	insoluble fraction	GO:0005626	25	1.7E-2

Pathway analysis with a curated collection was used to visualize and integrate transcriptomic data. Eight transcripts associated with angiogenesis were up-regulated (S1 Fig): angiotensin receptor type 2 (*agtr2*), transforming growth factor beta receptor type 3 (*tgfbr3*), fibroblast growth factor receptors type 2 and 4 (*fgfr2* and *fgfr4*), platelet-derived growth factor receptor type alpha (*pdgfra*), intracellular effectors related to hepatocyte proliferation: mitogen-activated protein kinase 6 (*mapk6*), proto-oncogene c-fos (*fos*), and transcription factor AP-1 (*jun*). Seventeen transcripts associated with fatty acid and cholesterol biosynthesis were down-regulated (S2 and S3 Figs, respectively): ATP-citrate synthase (*acly*), acetyl- carboxylase 1 (*acaca*), fatty acid synthase (*fasn*), hydroxyacyl-coenzyme A dehydrogenase (*hadh*), peroxisomal trans-2-enoyl- reductase (*pecr*), stearoyl-CoA desaturase 5 (*scd5*), long-chain-fatty-acid-ligase 3 (*acsl3*), 3-hydroxy-3-methylglutaryl-coenzyme A synthase 1 (*hmgcs1*), 3-hydroxy-3-methylglutaryl-CoA reductase (*hmgcr*), mevalonate decarboxylase (*mvd*), isopentenyl-diphosphate delta isomerase 1 (*idi1*), farnesyl diphosphate synthase (*fdps*), squalene epoxidase (*sqle*), lanosterol synthase (*lss*), cytochrome P450, family 51, subfamily A1 (*cyp51a1*), NAD(P)dependent steroid dehydrogenase-like (*nsdhl*), lathosterol oxidase (*sc5dl*), and 7-dehydrocholesterol reductase (*dhcr7*).

A general down-regulation was observed in the PPARα signaling pathway (S4 Fig), including the transcription factor, peroxisome proliferator-activated receptor alpha (*ppara*), and transcripts associated with ketogenesis (hydroxymethylglutaryl-CoA synthase, *hmgcs1*), lipid transport (apolipoprotein B-100, *apob*; apolipoprotein D, *apod*), cholesterol metabolism (sterol 26-hydroxylase, *cyp27a1*), fatty acid transport (fatty acid-binding protein 1, *fabp1*; fatty acid-binding protein 3, *fabp3*; long-chain-fatty-acid-CoA ligase 3, *acsl3*; and long-chain-fatty-acid-CoA ligase ACSBG2, *acsbg2*), and fatty acid beta oxidation (peroxisomal acyl-coenzyme A oxidase 3; *acox3*). An up-regulation was observed in the expression of two transcripts associated with fatty acid omega oxidation (alcohol dehydrogenase class-3, *adh5*, and cytochrome P450 2D6, *cyp2d6*). In addition, a down-regulation was observed in triglyceride synthesis (glycerol-3-phosphate acyltransferase 1, *gpam*; 1-acyl-sn-glycerol-3-phosphate acyltransferase gamma, *agpat3*; diacylglycerol O-acyltransferase 1, *dgat1*; and diacylglycerol O-acyltransferase 2, *dgat2*) and a consequent up-regulation in transcripts associated to triglyceride degradation (lipase member H, *liph* and patatin-like phospholipase domain-containing protein 2, *pnpla2*) (S5 Fig).

Transcripts related to gluconeogenesis and glycolysis pathways showed changes in gene expression (S6 Fig), including up-regulated glucose-6-phosphatase (*g6pc*), fructose-bisphosphate aldolase C (*aldoc*), and aspartate aminotransferase (*got1*) and down-regulated transcripts glucokinase (*gck*), alpha-enolase (*eno1*), pyruvate kinase (*pklr*), and dihydrolipoyl dehydrogenase (*dld*).

### RNA-seq validation

**Fig 2:**
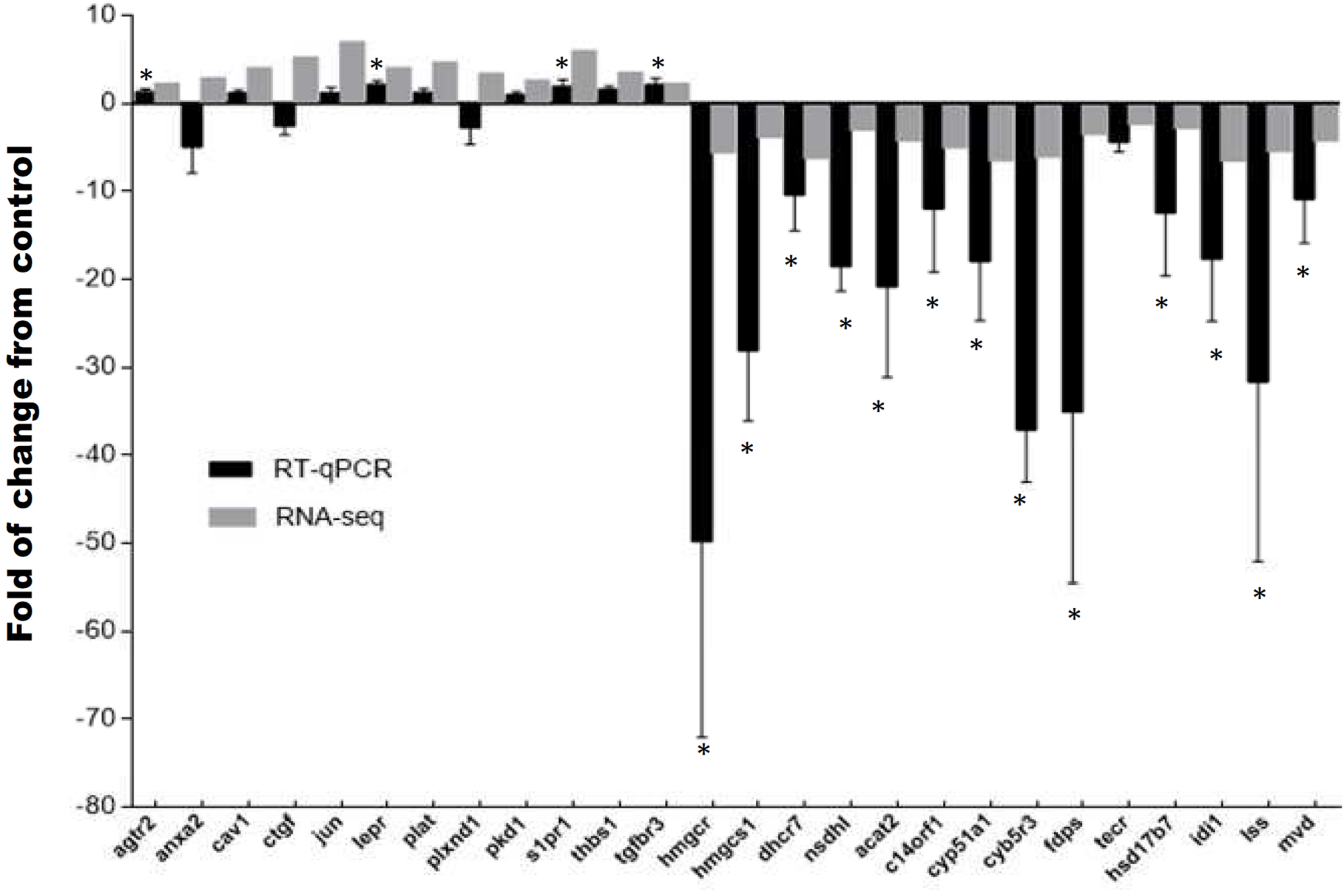
Quantitative real time PCR validation of differentially expressed transcripts. Expression of fold changes measured by RNA-seq and qPCR are indicated in the grey and black columns, respectively. *agtr2* (type-2 angiotensin ii receptor), *anxa2* (annexin a2), *cav1* (caveolin-1), *ctgf* (connective tissue growth factor precursor), *jun* (transcription factor ap-1), *lepr* (leptin receptor precursor), *plat* (tissue-type plasminogen activator precursor), *plxnd1* (plexin-b2 precursor), *pkd1* (polycystin-1 precursor), *s1pr1* (sphingosine 1-phosphate receptor 1), *thbs1* (thrombospondin-2 precursor), *tgfbr3* (transforming growth factor beta receptor type 3 precursor), *hmgcr* (3-hydroxy-3-methylglutaryl-coenzyme a reductase), *hmgcs1* (hydroxymethylglutaryl- cytoplasmic), *dhcr7* (7-dehydrocholesterol reductase), *nsdhl* (sterol-4-alpha-carboxylate 3- decarboxylating), *acat2* (acetyl-CoA acetyltransferase), *c14orf1* (ergosterol biosynthetic protein), *cyp51a1* (lanosterol 14-alpha demethylase), *cyb5r3* (nadh-cytochrome b5 reductase 3), *fdps* (farnesyl pyrophosphate synthase), *tecr* (trans- enoyl- reductase), *hsd17b7* (3-keto-steroid reductase), *idi1* (isopentenyl-diphosphate delta-isomerase 1), *lss* (lanosterol synthase), *mvd* (diphosphomevalonate decarboxylase), *fau* (40S ribosomal protein). Significant differences between the control and stressed groups are shown as * (P < 0.05) and ** (P < 0.01).

To validate the RNA-seq analysis, we used qPCR to assay stress-associated changes in mRNA levels of 26 genes. We selected 12 transcripts from the first functional annotation up-regulated cluster (*agtr2*, *anxa2*, *cav1*, *ctgf*, *jun*, *lepr*, *plat*, *plxnd1*, *pkd1*, *s1pr1*, *thbs1*, *tgfbr3*) and 14 transcripts from the first functional annotation down-regulated cluster (*hmgcr*, *hmgcs*, *dhcr7*, *nsdhl*, *acat2*, *c14orf1*, *cyp51a1*, *cyb5r3*, *fdps*, *tecr*, *hsd17b7*, *idi1*, *lss*). The transcript expression fold-changes measured by qPCR and RNA-seq had a high statistical correlation (R^2^ = 0.78, P < 0.001) (Fig 2).

### Hepatotoxicity induced by handling-stress

**Fig 3:**
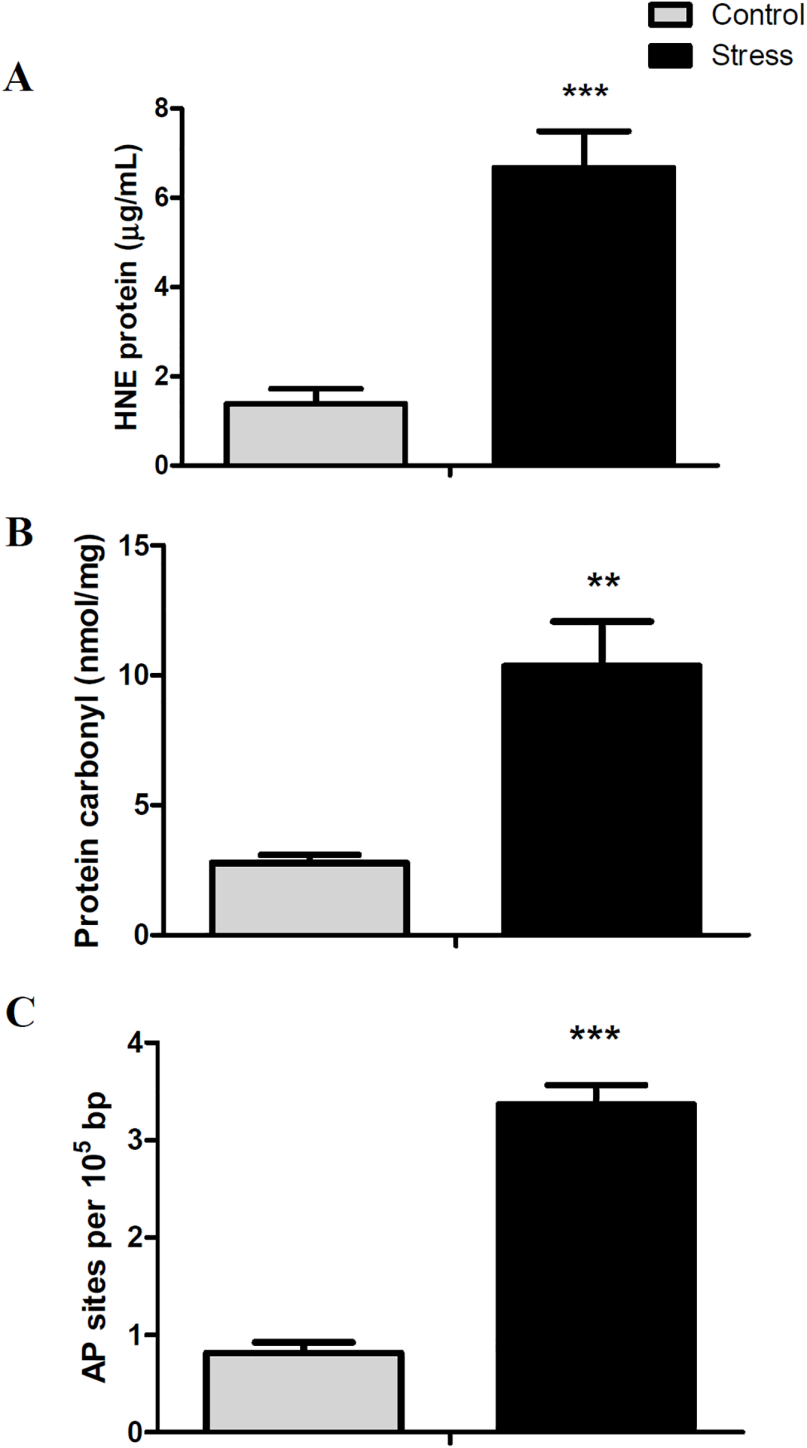
Hepatic levels of lipid peroxidation, protein carbonylation and DNA oxidative damage. All data are represented as means ± SEM (n = 4). Significant differences between control and stressed groups are shown as * (P < 0.05) and ** (P < 0.01).

**Fig 4:**
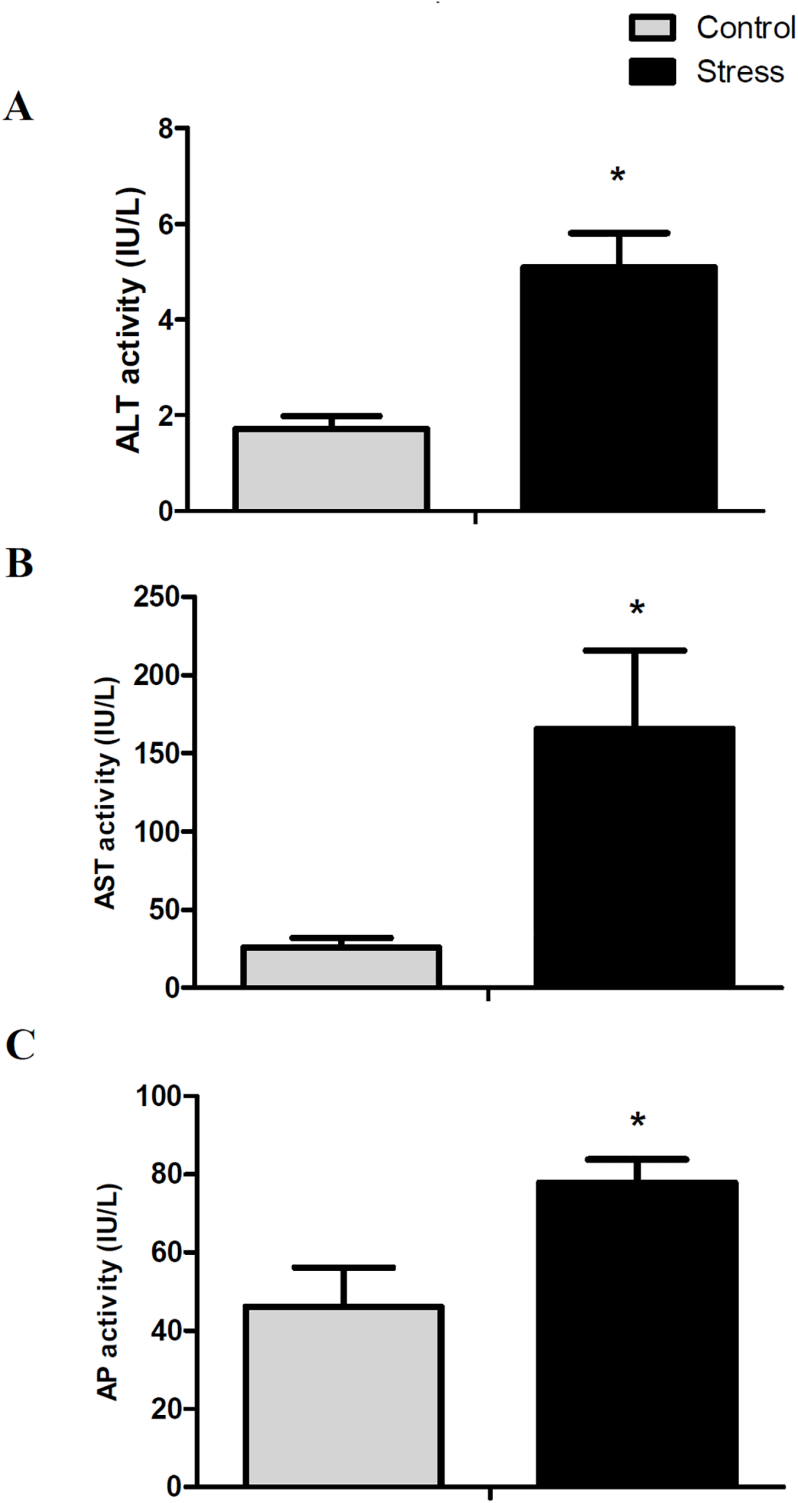
Plasmatic levels of ALT (alanine aminotransferase), AST (aspartate aminotransferasa), and AP (alkaline phosphatase). All data are represented as means ± SEM (n = 4). Significant differences between control and stressed groups are shown as * (P < 0.05) and ** (P < 0.01).

To evaluate the hepatotoxicity induced by this stress, we measured cellular and plasmatic markers of liver damage. Handling-stress significantly increased the rate of lipid peroxidation (Fig 3A), protein carbonylation (Fig 3B), and DNA oxidative damage (Fig 3C) (3.4-fold, 2.8-fold, and 3.3-fold, respectively) in comparison to control conditions. Similarly, the plasmatic concentrations of alanine aminotransferase (Fig 4A), aspartate aminotransferasa (Fig 4B) and alkaline phosphatase (Fig 4C) were significantly raised in stressed animals (1.3-fold, 3.8-fold, and 1.8-fold, respectively), in comparison to control conditions. In addition, histological liver analysis reveals that hepatocytes from control conditions have the typical polygonal cell shape and clear cytoplasm. However hepatocytes from stressed conditions start to lose their polygonal shape and have a condensed cytoplasm characteristics of liver fibrosis (S7 Fig).

## Discussion

Our results are the first to describe the molecular and biochemical hepatic response to stress in the marine teleost, the red cusk-eel. This species’ molecular response to stress has been documented in terms of the effects of cortisol-induced stress in skeletal muscle atrophy [24]. This work provides new evidence of the susceptibility of this non-domesticated organism and reinforces the idea that each species has specificities in their stress tolerance. Here, RNA-seq analysis evaluated the hepatic transcriptomic response to handling-stress, revealing a differential gene expression primarily associated with biological pathways, such as angiogenesis, fatty acid biosynthesis, cholesterol biosynthesis, PPARα signaling, triglyceride metabolism, gluconeogenesis and glycolysis. The results obtained by this *in silico* approach were validated by qPCR analysis of representative cholesterol and angiogenesis pathway genes, as well as the assessment of hepatic metabolites and markers of hepatic damage.

### Stress and glucose metabolism

Numerous studies in teleost demonstrate that stress is intimately related to glucose metabolism, because its oxidation meets the increased energy demand needed to cope with stress [30,31]. Studies in rainbow trout (*O*. *mykiss)* have determined that handling-stress induces an elevation in the expression of key enzymes involved in gluconeogenesis during the recovery phase, including phosphoenolpryruvate carboxykinase (*pck1*) and glucose-6-phosphatase (*g6pc*), as well as a decrease in the expression of pyruvate kinase (*pklr*) and glucokinase (*gck*) involved in glycolisis [32,33]. Most expressions of these genes are directly regulated by the glucocorticoid receptor [9,14]. Consistent with these observations, handling-stress in the red cusk-eel induced an up-regulation in the expression of gluconeogenic gene *g6pc* and a down-regulation of glycolitic genes *pklr* and *gck*. These results are similar to those of juvenile carp (*Cyprinus carpio*) and Mozambique tilapia (*Oreochromis mossambicus*), for which the administration of a high dose of cortisol significantly increased glucose serum levels and glucose-6 phosphatase activity, revealing a major role for cortisol in gluconeogenesis [34,35]. Similar to our observations, chronic cortisol administration in gilthead sea bream (*Sparus aurata*), induces a down-regulation of enolase 1 (*eno1*) [18], a glycolitic gene. In addition, in Senegalese sole (*Solea senegalensis*), repeated handling-stress induces a decrease of enolase 1 protein expression [36]. Therefore, we hypothesize that the increase in plasma glucose levels seen in the red cusk-eel after stress may be associated with enhanced muscle protein catabolism necessary for liver gluconeogenesis, even though increases in plasmatic levels of lactate during stress were not found [24]. The increase in plasmatic glucose levels can also be related to glycogen hydrolysis (glycogenolysis), evidenced by its decrease in the liver. Indeed, stress and cortisol have been demonstrated to play a fundamental role in glycogen metabolism in rainbow trout [37,38], although we did not find changes in the expression of genes associated with such catabolism.

### Stress and lipid metabolism

The liver also plays a central role in lipid metabolism [39], and in the current study, handling-stress induced a general down-regulation in the cholesterol and fatty acid biosynthesis pathways. The stress-induced inhibition of cholesterol biosynthesis is in line with previous data on rainbow trout, where plasma cholesterol levels decreased after 30 days of high stocking densities [40]. Additionally, the down-regulation in fatty acid biosynthesis is concordant with observations of rainbow trout following handling-stress, in which fatty acid synthase (*fasn*) expression and activity decreased [37].

It is well known that under stressful conditions another metabolic fuel, ketone bodies, is generated through NEFA beta oxidation[40]. A major NEFA source is the hydrolysis of liver and adipose tissue triglycerides through lipase-catalyzed lipolysis [41]. Consistent with this, handling-stress induced an increase in the expression of *liph* and *pnpla2* lipases as well as, a general down-regulation in the expression of genes associated with triglyceride synthesis. Lipase H (LIPH) is an evolutionary conserved phospholipase which catalyzes the production of fatty acids and lysophosphatidic acid (LPA) [42]. PNPLA2 is a member of the patatin-like phospholipase domain-containing protein family, which catalyzes the initial step in triglyceride hydrolysis [43]. Although there are reports of the over-expression of both genes in mammal tissues under pathophysiological conditions [44], no reports exist linking these genes to a stress response in teleosts. Consistent with the over-expression of *liph* and *pnpla2* under stressful conditions, there was a decrease in triglyceride levels and an increase in NEFA hepatic levels. The dramatic increases in hepatic NEFA levels may be associated with enhanced triglyceride hydrolysis and impaired fatty acid beta oxidation, similar to decreases seen in hepatic triglyceride levels in rainbow trout caused by acute handling-stress [37]. It is therefore probable that increased hepatic fatty acid accumulation is related with the development of liver steatosis [45].

### Stress and liver steatosis

Liver steatosis is defined as an accumulation of excessive triglycerides and other fats inside liver cells [46], although fish that are fed diets rich in unsaturated fatty acids are known to develop this condition [47], there are no reports relating this condition to stress. A direct relationship between glucocorticoids and fatty liver disease is described in mammals, with glucocorticoids acting as a promoter of lipid accumulation, leading to inflammation, and the potential for fibrosis [45]. This accumulation in hepatocyte diminishes mitochondrial oxidative capacity, further reducing the state of the electron transport chain complexes and stimulating peroxisomal and microsomal pathways for omega oxidation [45]. The consequent increased generation of reactive oxygen species (ROS) and reactive aldehydic derivatives causes oxidative stress [46].

We found increased expressions of cytochrome P450 2D6 (*cyp2d6*) and alcohol dehydrogenase class-3 (*adh5*), genes related to fatty acid omega oxidation and a consistent increase in lipid peroxidation, protein carbonylation, and DNA oxidation induced by stress. While there are no reports in teleost that link the fatty acid accumulation in liver to oxidative damage, it has been recently reported in tapertail anchovy (*Coilia nasus*) that loading stress induces lipid peroxidation, expression of apoptosis-associated markers, and increased plasmatic levels of ALT and AST [48]. While the authors associated the liver damage to TNFα-induced apoptosis, interestingly they also reported an increase of lipid metabolic genes, similar to our observations.

Recently, a study developed with a mammalian model of hepatocellular steatosis established the effects of free fatty acid and the mechanism associated to oxidative stress [49]. The authors reported that high concentration of free fatty acid induced excessive lipid accumulation and oxidative stress by down-regulating the *ppara* expression, similar to our observations [49]. PPARα is nuclear hormone receptors, and regulates the expression of many genes involved in fatty acid oxidation, ketogenesis, gluconeogenesis, cholesterol catabolism, among others. In fish, PPARα have been identified and characterized in several species however there are no direct reports that associate changes in its expression during stress response[50]. However, the systemic administration of the PPARα antagonist, WY-14643, has been shown to induce a decrease in NEFA plasmatic levels associated to increased fatty acid beta oxidation in turbot (*Scophthalmus maximus*) [51]. Therefore, we speculate that stress-impaired PPARα expression should induce an increase in NEFA overload and cytochrome P450-dependent-omega oxidation. Consistent with these observations, a direct role for glucocorticoids in the increase of TG hydrolysis, as well as in the inhibition of beta oxidation and cholesterol biosynthesis, has been demonstrated in mammals through *in vitro* and *in vivo* trials [52,53].

## Conclusions

Handling-stress induced major changes in the red cusk-eel hepatic metabolic response. Under stress, 259 genes, mostly associated to angiogenesis, gluconeogenesis, and triglyceride degradation, were up-regulated. Conversely, 293 genes, associated to PPARα signaling, fatty acid biosynthesis, cholesterol biosynthesis, and glycolysis, were down-regulated. These transcriptomic analyses are supported by quantifications of liver metabolites, oxidative damage, markers of hepatic damage and liver histology. This is the first study to suggest that handling-stress is powerful inducer of liver steatosis in a teleost, providing valuable information for monitoring the culturing and growth of marine fish species under intensive rearing conditions.

## Supporting information

S1 Table Primer sequences for qPCR assay, amplicon size, and PCR efficiencies for genes used in the study. Sequences were derived from the red cusk-eel reference transcriptome.
(XLSX)

S2 Table Complete list of up-regulated and down-regulated hepatic transcripts in response to handling-stress. (XLSX)

S1 Fig Illustration created using Pathvisio v3 of the angiogenesis pathway involved in the hepatic stress response based on RNA-seq expression analysis. The red colors indicate an increase in any of the components of the pathways.
(TIF)

S2 Fig Illustration created using Pathvisio v3 of the fatty acid biosynthesis pathway involved in the hepatic stress response, based on RNA-seq expression analysis. The green colors indicate a decrease in any of the components of the pathways.
(TIF)

S3 Fig Illustration created using Pathvisio v3 of the cholesterol biosynthesis pathway involved in the hepatic stress response, based on RNA-seq expression analysis. The green colors indicate a decrease in any of the components of the pathways.
(TIF)

S4 Fig Illustration created using Pathvisio v3 of the PPARα pathway involved in the hepatic stress response, based on RNA-seq expression analysis. The green and red colors indicate a decrease and increase in any of the components of the pathways, respectively.
(TIF)

S5 Fig Illustration created using Pathvisio v3 of the triglyceride pathway involved in the hepatic stress response, based on RNA-seq expression analysis. The green and red colors indicate a decrease and increase in any of the components of the pathways, respectively.
(TIF)

S6 Fig Illustration created using Pathvisio v3 of the glycolisis and gluconeogenesis pathways involved in the hepatic stress response, based on RNA-seq expression analysis. The green and red colors indicate a decrease and increase in any of the components of the pathways, respectively.
(TIF)

S7 Fig Histology of red cusk-eel liver at A) control and B) stressed conditions (n = 4, per group). The samples were stained with hematoxylin/eosin, observed in an Olympus BX-61 microscope at 100X and photographed with a Leica DF300 camera.
(TIF)
