# Supplementary figures and images for: Transcriptomic analysis of the hepatic response to stress in the red cusk-eel (Genypterus chilensis): Insights into lipid metabolism, oxidative stress and liver steatosis
Source: PLoS One. 2017 Apr 27;12(4):e0176447. doi: 10.1371/journal.pone.0176447 (PMC5407771; doi:10.1371/journal.pone.0176447)

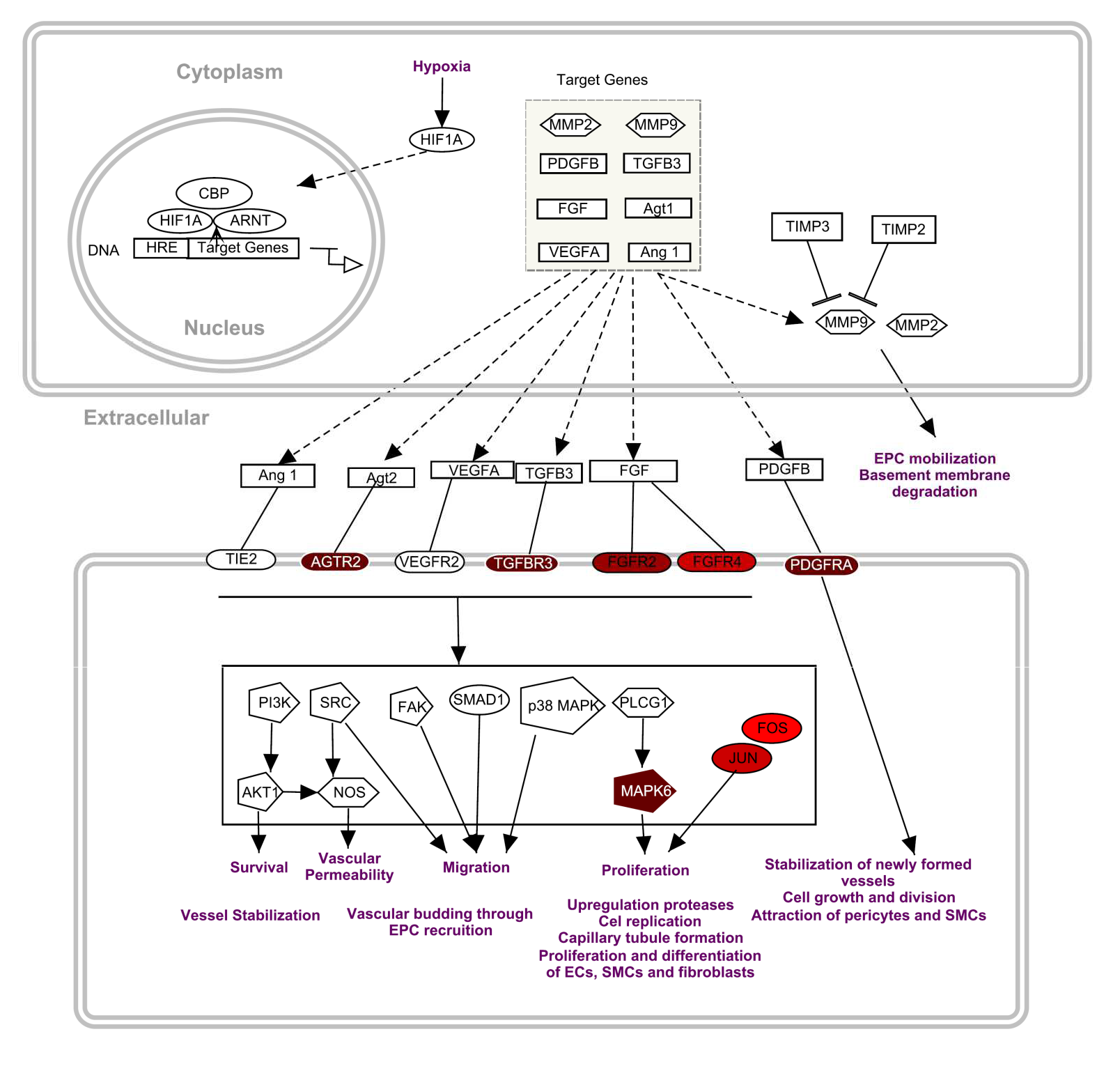

Supplement: S1 Fig — The red colors indicate an increase in any of the components of the pathways. (TIF) [file pone.0176447.s003.tif]

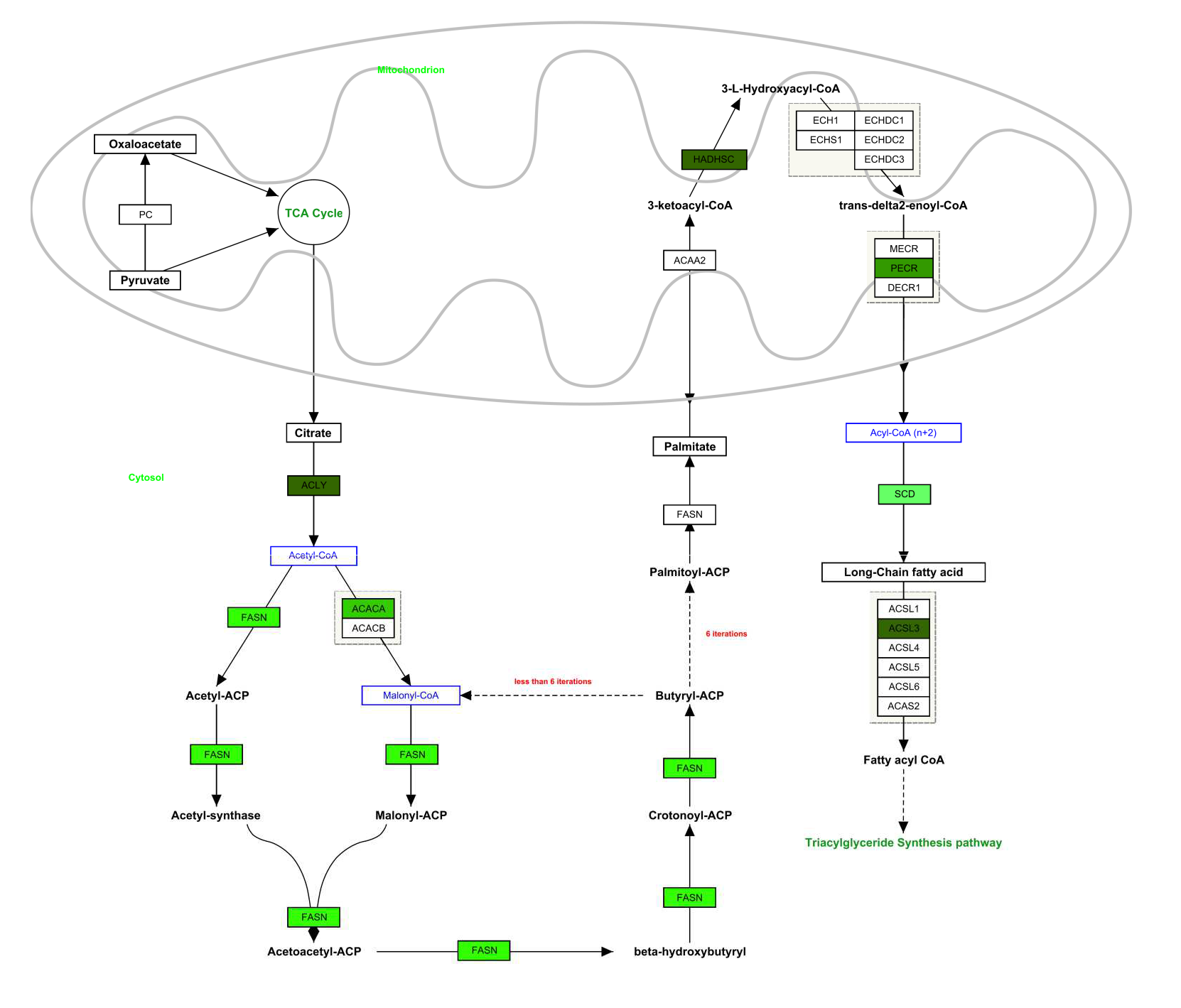

Supplement: S2 Fig — The green colors indicate a decrease in any of the components of the pathways. (TIF) [file pone.0176447.s004.tif]

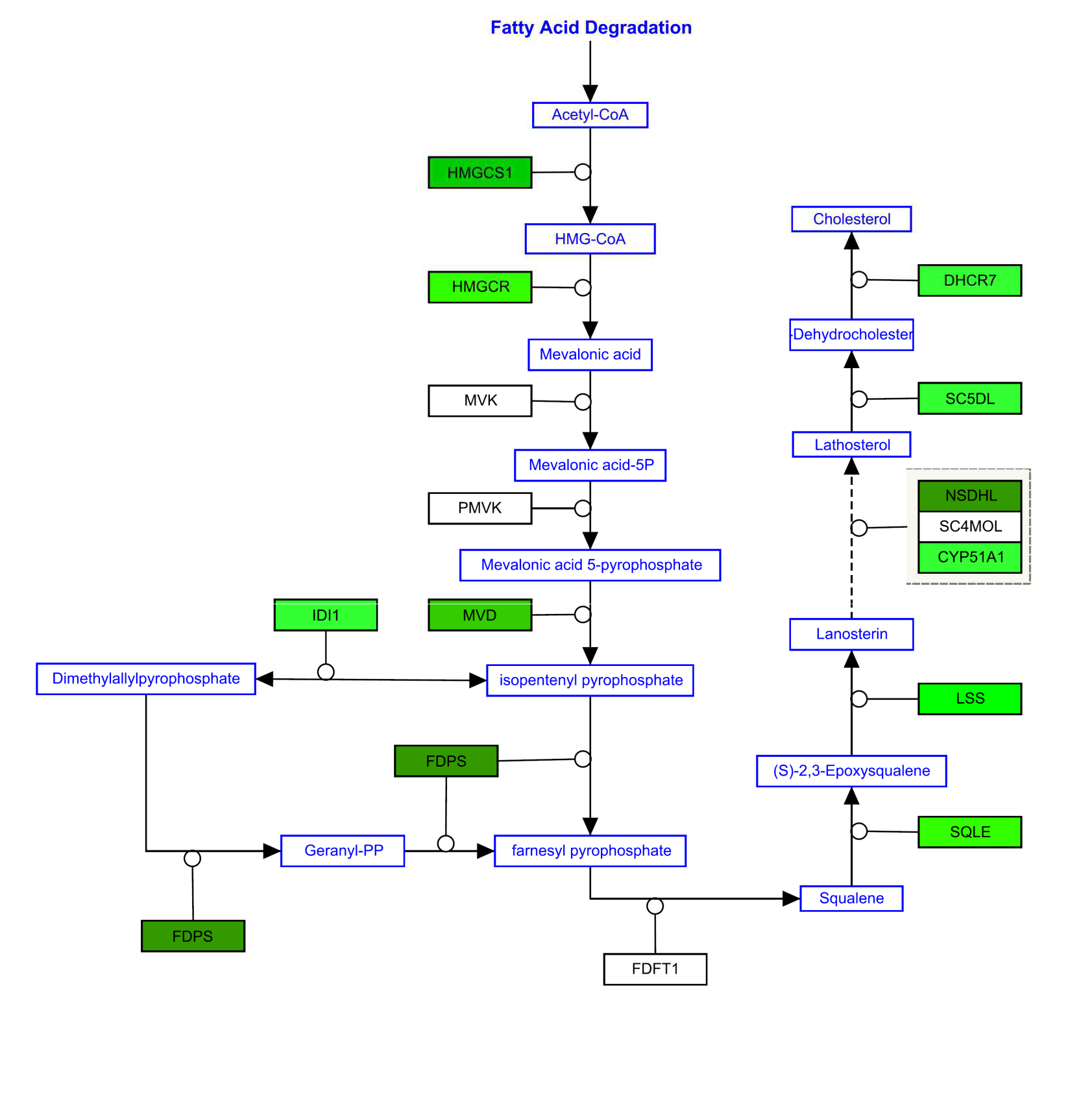

Supplement: S3 Fig — The green colors indicate a decrease in any of the components of the pathways. (TIF) [file pone.0176447.s005.tif]

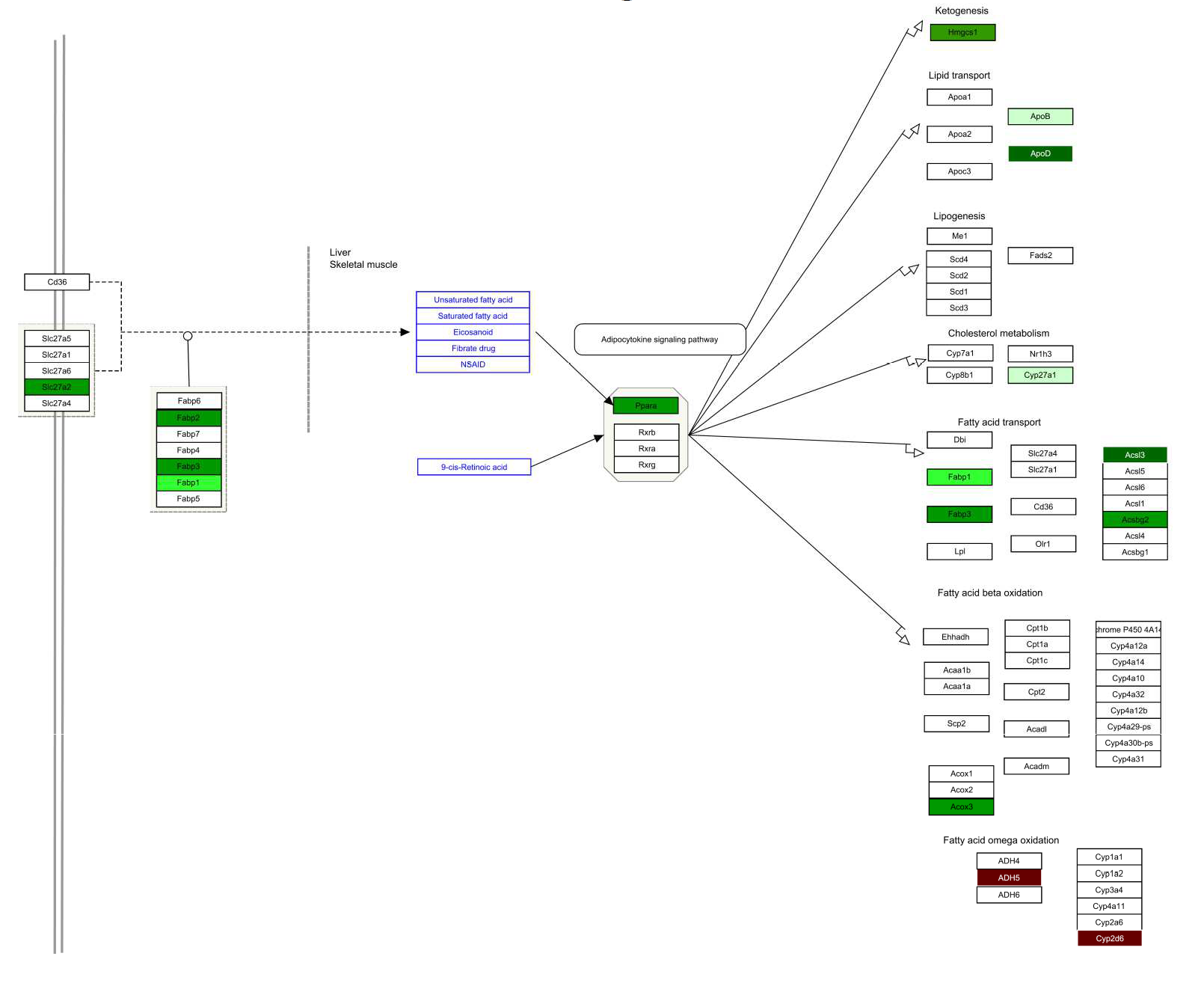

Supplement: S4 Fig — The green and red colors indicate a decrease and increase in any of the components of the pathways, respectively. (TIF) [file pone.0176447.s006.tif]

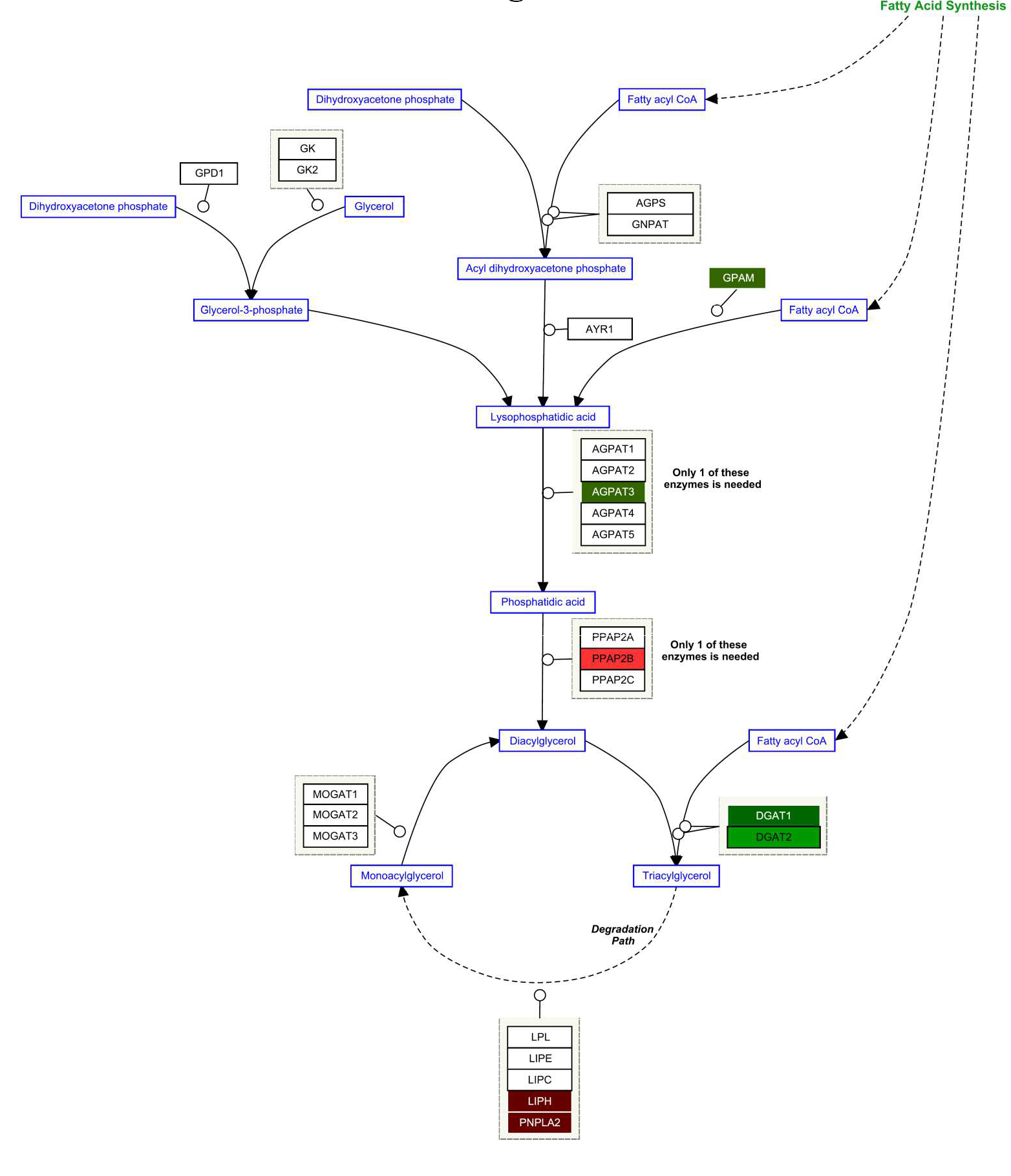

Supplement: S5 Fig — The green and red colors indicate a decrease and increase in any of the components of the pathways, respectively. (TIF) [file pone.0176447.s007.tif]

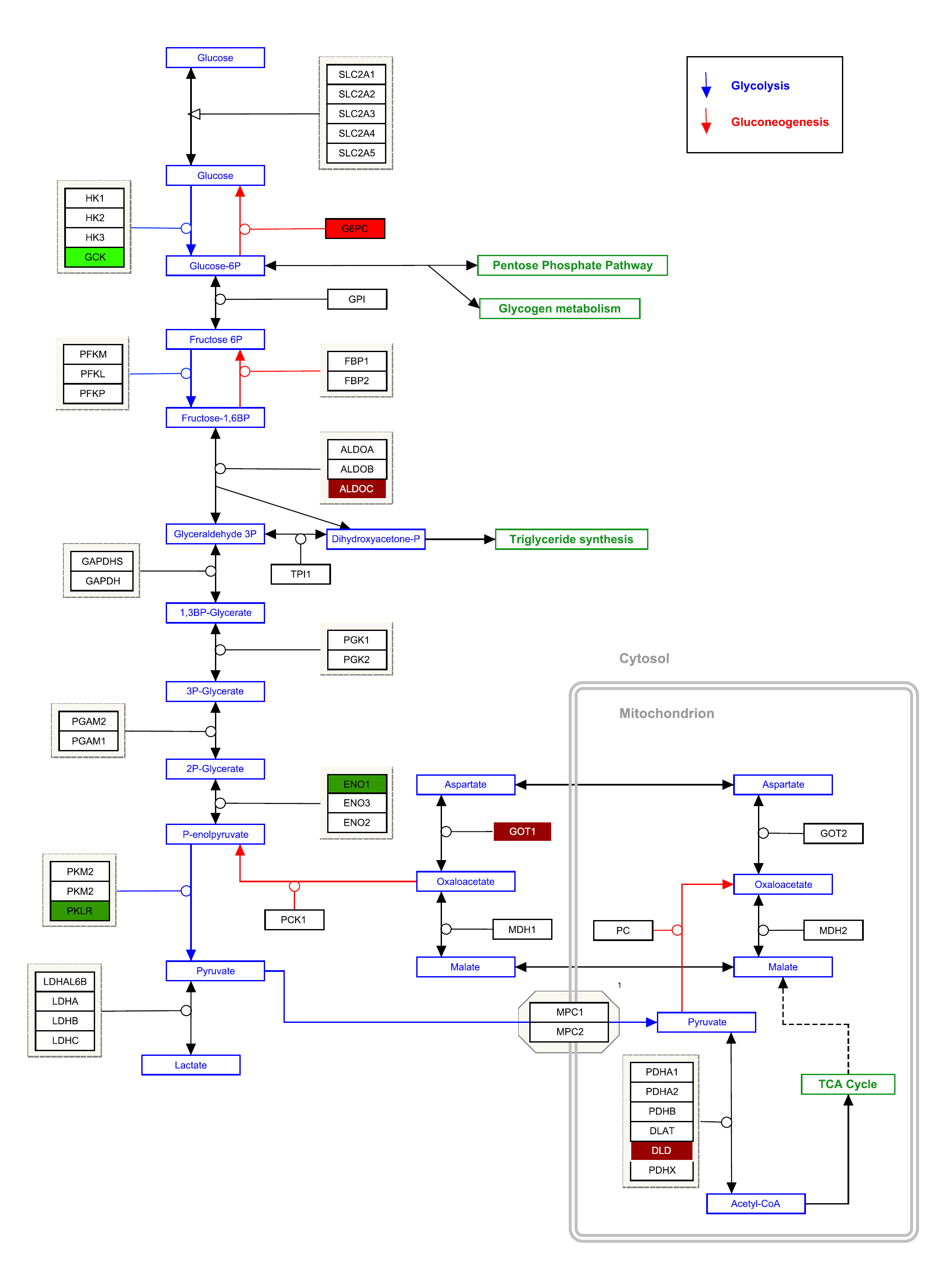

Supplement: S6 Fig — The green and red colors indicate a decrease and increase in any of the components of the pathways, respectively. (TIF) [file pone.0176447.s008.tif]

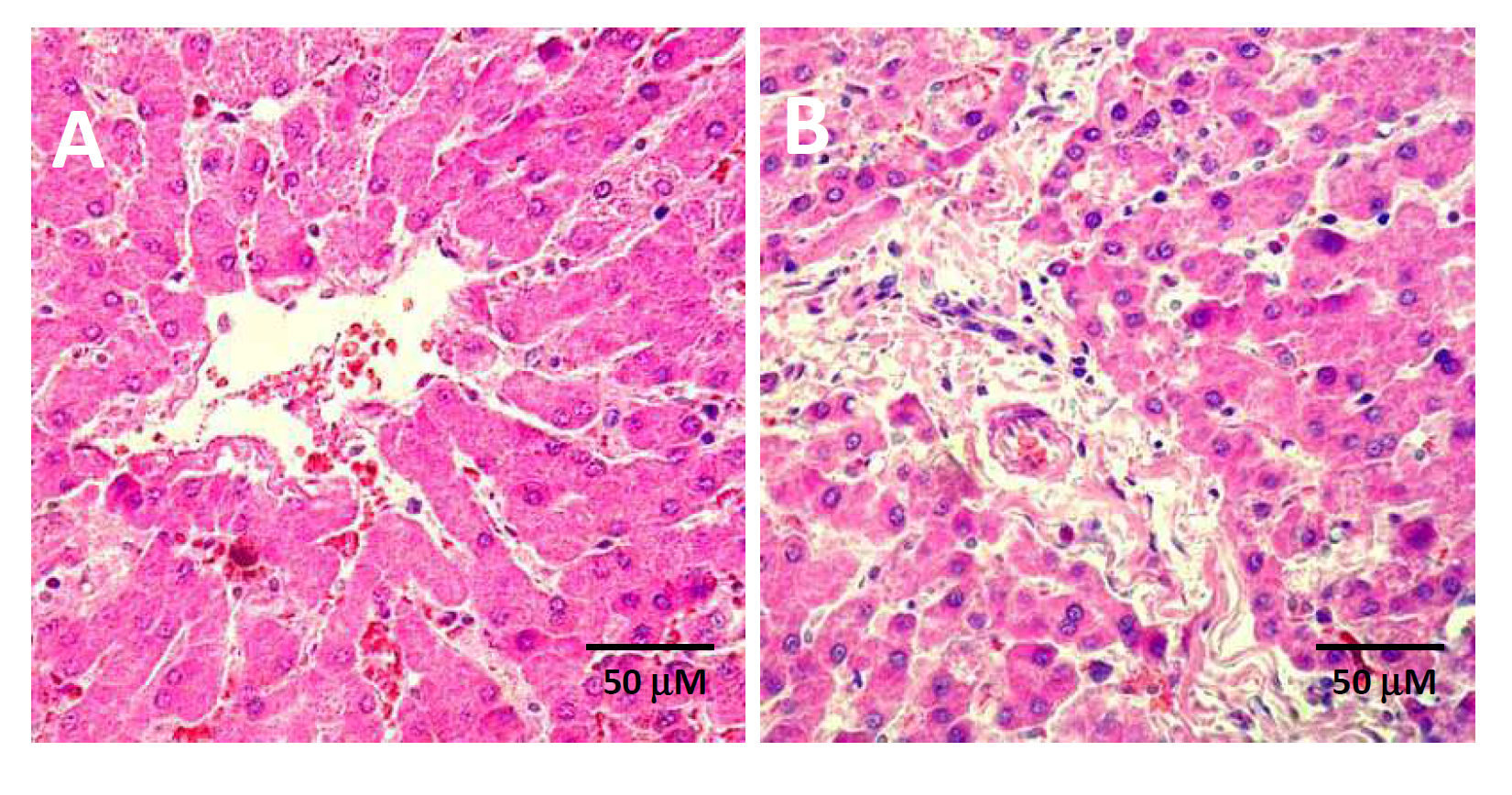

Supplement: S7 Fig — Histology of red cusk-eel liver at A) control and B) stressed conditions (n = 4, per group). The samples were stained with hematoxylin/eosin, observed in an Olympus BX-61 microscope at 100X and photographed with a Leica DF300 camera. (TIF) [file pone.0176447.s009.tif]
